# Results of a confirmatory mapping tool for *Lymphatic filariasis* endemicity classification in areas where transmission was uncertain in Ethiopia

**DOI:** 10.1371/journal.pntd.0006325

**Published:** 2018-03-26

**Authors:** Heven Sime, Katherine M. Gass, Sindew Mekasha, Ashenafi Assefa, Adugna Woyessa, Oumer Shafi, Kadu Meribo, Biruck Kebede, Kisito Ogoussan, Sonia Pelletreau, Moses J. Bockarie, Amha Kebede, Maria P. Rebollo

**Affiliations:** 1 Ethiopian Public Health Institute, Addis Ababa, Ethiopia; 2 The Task Force for Global Health, Atlanta Georgia, United States of America; 3 Federal Ministry of Health, Addis Ababa, Ethiopia; 4 Centers for Disease Control and Prevention, Atlanta, Georgia, United States of America; 5 European & Developing Countries Clinical Trials Partnership (EDCTP), Cape Town, South Africa; Emory University, UNITED STATES

## Abstract

**Background:**

The goal of the global *lymphatic filariasis* (LF) program is to eliminate the disease as a public health problem by the year 2020. The WHO mapping protocol that is used to identify endemic areas in need of mass drug administration (MDA) uses convenience-based sampling. This rapid mapping has allowed the global program to dramatically scale up treatment, but as the program approaches its elimination goal, it is important to ensure that all endemic areas have been identified and have received MDA. In low transmission settings, the WHO mapping protocol for LF mapping has several limitations. To correctly identify the LF endemicity of woredas, a new confirmatory mapping tool was developed to test older school children for circulating filarial antigen (CFA) in settings where it is uncertain. Ethiopia is the first country to implement this new tool. In this paper, we present the Ethiopian experience of implementing the new confirmatory mapping tool and discuss the implications of the results for the LF program in Ethiopia and globally.

**Methods:**

Confirmatory LF mapping was conducted in 1,191 schools in 45 woredas, the implementation unit in Ethiopia, in the regions of Tigray, Amhara, Oromia, SNNP, Afar and Harari, where the results of previous mapping for LF using the current WHO protocol indicated that LF endemicity was uncertain. Within each woreda schools were selected using either cluster or systematic sampling. From selected schools, a total of 18,254 children were tested for circulating filarial antigen (CFA) using the immuno-chromatographic test (ICT).

**Results:**

Of the 18,254 children in 45 woredas who participated in the survey, 28 (0.16%) in 9 woredas tested CFA positive. According to the confirmatory mapping threshold, which is ≥2% CFA in children 9–14 years of age, only 3 woredas out of the total 45 had more CFA positive results than the threshold and thus were confirmed to be endemic; the remaining 42 woredas were declared non-endemic. These results drastically decreased the estimated total population living in LF-endemic woredas in Ethiopia and in need of MDA by 49.1%, from 11,580,010 to 5,893,309.

**Conclusion:**

This study demonstrated that the new confirmatory mapping tool for LF can benefit national LF programs by generating information that not only can confirm where LF is endemic, but also can save time and resources by preventing MDA where there is no evidence of ongoing LF transmission.

## Author summary

*Lymphatic filariasis* (LF) is a mosquito-borne parasitic disease, caused by 3 nematode parasites, *Wuchereria bancrofti*, *Brugia malayi and Brugia timori*. The aim of the Global Program to Eliminate LF (GPELF) is to interrupt LF transmission through mass drug administration (MDA) by 2020 and to alleviate the suffering of affected people. Mapping is the first programmatic step to determining areas of LF endemicity and establishing a national program. Ethiopia was believed to be endemic for LF, but until recently the distribution of LF in the country was unknown. From 2008–2013, mapping for LF was conducted using the current WHO protocol, and 112 woredas were identified as endemic or possibly endemic. In 45 of these 112 woredas, only a single CFA positive result was found (<1% prevalence), which called into question the stutus of transmission and need for MDA. To help resolve this uncertainty, a new confirmatory mapping tool was designed and tested in Ethiopia. The new mapping tool was piloted in the 45 woredas with uncertain LF transmission from the 2008–2013 mapping (S1 Table). This mapping confirmed that only 3 of the 45 woredas were endemic, which decreased estimated total population at risk of LF and in need of MDA from 11,580,010 to 5,893,309.

## Introduction

Lymphatic Filariasis (LF) is a neglected tropical disease (NTD), known to disproportionally cause disability among people with low socioeconomic status living in endemic areas [1]. Globally, about 1.4 billion people are at risk of LF infection[2]. Though the disease is endemic in 73 countries, 80% of the total number of people at risk live in only 10 countries [3]. LF is caused by three types of nematode parasites: *Wuchereria bancrofti*, *Brugia malayi* and *Brugia timori*[4]. More than 90% of human LF is caused by *Wuchereria bancrofti*, nine percent is caused by *Brugia malayi* in southeast and eastern Asia, and the remaining 1% is caused by *Brugia timori* in the Pacific region [5,6]. In Africa, *Wuchereria bancrofti* is the only known cause of LF[7].

Preventive chemotherapy (PC) for LF is a single dose of albendazole (ALB), given in combination with either diethylcarbamazine (DEC) or, in countries where onchocerciasis is co-endemic, ivermectin (IVM) [5,8]. In at risk area the eligible population is treated through annual mass drug administration (MDA). Five years of MDA with combination PC can result in a 99% reduction in microfilaria prevalence[9]. The World Health Organization (WHO) has established a global goal of eliminating LF by 2020 using a strategy of MDA with PC for eligible individuals living in endemic areas.

From 2008–2013, Ethiopia was mapped for LF using the current WHO mapping protocol for LF. The protocol uses two-stage cluster sampling and convenience sampling of adults to determine LF endemicity and treatment need at the woreda level. First, two communities per woreda are purposively selected using information obtained from woreda health bureau. In each selected community, a convenience sample of 100 individuals of age greater than 15 years, with equal sex proportion, are selected from a random starting point. Each individual is tested for circulating filarial antigen using immunochromatographic test (ICT). A woredas is classified as endemic and in need of MDA when the prevalence of CFA is 1% or greater [10].

The Faculty of Medicine at Addis Ababa University conducted the first LF mapping survey from 2008 to 2012 in 112 woredas using the current WHO mapping protocol [11]. An additional 4 woredas were surveyed in 2012. In 2013, the Ethiopian Public Health Institute (EPHI) and partners surveyed an additional 658 woredas, again using the WHO mapping protocol. As a result of these surveys 774 woredas, 93% of the total number of woredas in the country, were mapped for LF by 2013 [12].

**Fig 1:**
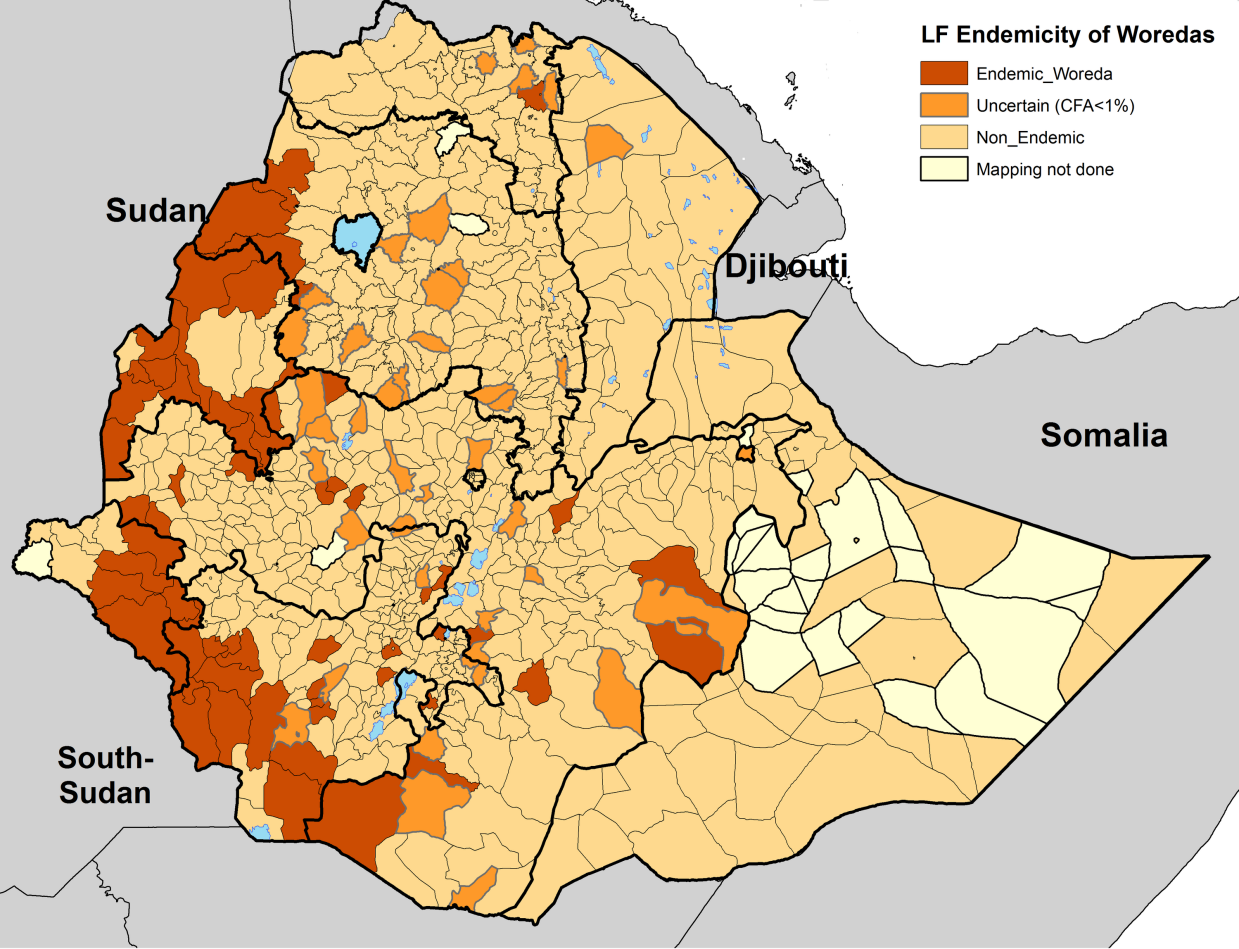
Map showing LF-endemicity status of woredas in Ethiopia. Endemic woredas are shown in Brown. Uncertain 45 woredas with <1% CFA+ shown in Orange. Non-Endemic woredas shown in Yellow. Unmapped 58 woredas shown in greyish_yellow color. www.gadm.org/ is the source of the administrative boundaries.

This mapping indicated that LF was endemic and potentially endemic in 112 woredas, with approximately 11 million people at risk as shown in Fig 1 [11,12]. According to WHO guidelines, if the prevalence of microfilaremia or CFA is 1% in either of the two sites, the woreda is classified as endemic and MDA is required [10]. However, 45 of the 112 woredas had only one CFA positive individual among the 200 tested, which is less than the 1% threshold. Such few positive cases indicate that these woredas are likely areas with very low or no transmission of LF and raised a question of whether MDA was warranted [12].

To address this concern of uncertain endemicity, a new LF confirmatory mapping tool was developed by global LF experts and approved during the 2014 meeting of the WHO’s Regional Program Review Group for Africa (RPRG)[13] for piloting in Ethiopia and Tanzania. The new confirmatory mapping tool was designed to be more precise than the current WHO LF mapping protocol. In contrast to the current WHO protocol, the confirmatory mapping tool aims to be more geographically representative at the woreda level and tests children, not adults. Unlike the Transmission Assessment Surveys in young children that are used to make treatment stopping decisions for LF, this confirmatory mapping methodology tests older children who have a longer period of potential exposure to LF infection. The new confirmatory methodology set a threshold for confirming LF endemicity and initiating MDA of >2% CFA in older children based on the null hypothesis that the average prevalence of antigenemia in older school children is >2%.

To operationalize this threshold for programmatic use, a critical cutoff was established such that the upper 1-sided 95% confidence interval does not exceed 2%. Using this cutoff value, MDA is not warranted if the total number of children testing positive for CFA in a district is less than or equal to the critical cutoff. However, when the number of children testing positive for CFA is greater than the critical cutoff, the woreda is considered endemic and requiring MDA.

Based on the number of primary schools in a woreda, either a cluster or systematic element sampling strategy is used. In woredas with 40 or more schools, cluster sampling is used. Thirty primary schools are randomly selected from a complete list of schools in the woreda using probability proportional to estimated size sampling (PPS) based on the total school enrollment rate, target sample size per school and assuming a 15% absentee rate. In each selected school, students with parental or guardian consent are sampled from grades in which the majority of children are between the age of 9 and 14.

In woredas with fewer than 40 schools, systematic element sampling is used, and all schools are selected for a total sample size of 320 children in the targeted grades. The task Force for Global Health built an electronic platform, Survey Sample Builder, which automated the sampling selection. The complete sampling design and statistical calculations of the confirmatory mapping methodology are described in Gass et al.[14].

In this paper, we present the Ethiopian experience of implementing the LF confirmatory mapping tool for the first time in 45 woredas of uncertain endemicity. We describe the logistics and feasibility of implementing this new mapping strategy and discuss the impact of the results on Ethiopia’s national LF program.

## Methods

### Study sites and participant characteristics

The confirmatory mapping protocol was piloted in 1,191 schools in 45 woredas where the results of 2013 mapping using the current WHO protocol indicated that LF endemicity was uncertain. Of the 45 woredas, 4 were in Tigray, 13 in Amhara, 19 in Oromia, 7 in SNNP, one in Afar and one in Harari regions.

Cluster sampling was performed in 27 large woredas, while systematic sampling was conducted in the remaining 18 woredas. In the 27 woredas where cluster sampling was used (≥40 schools), the target sample size was 480 students per woreda. Within each school, students were selected according to a defined sampling interval based on the total number of students in the targeted grades in the school. When a selected school had fewer students in the targeted grades (according to the enrollment figures), the school was merged with another nearby school.

In the 18 woredas where systematic sampling was used (<40 schools), the target sample size was 320 students per woreda. In this case, all schools in the woreda were visited, and students in the targeted grades were included after accounting for the expected absentee rate. The sampling interval was similar in each school.

With the exception of a few woredas, sampling took place in all initially selected schools (S1 Table). Of the 1,227 schools selected, data collection was conducted in 97% (n = 1,191) of them. In 3% (n = 36) of the schools, data collection could not be conducted due to security issues and scarcity of water which had closed the schools during the time of data collection. The school enrollment rate obtained from Ethiopian Federal Ministry of Education was very close to the actual school enrollment rate in 33 of the total 45 woredas.

### Field work

In advance of the survey, the national team at EPHI contacted the regional health bureaus with an official letter requesting health professionals to participate as survey team members. Sixty-one persons (42 laboratory technicians and 19 nurses) from the regions where the survey was conducted (Tigray, Oromia, Amhara, SNNPR, Afar and Harari) were identified and sent to EPHI in Addis Ababa to participate in training on the sampling methodology and sample collection and processing. The three-day training was composed of theoretical and practical sessions. The theoretical session addressed how to communicate the confirmatory mapping strategy to community and school leaders and how they, in turn, can coordinate and mobilize students. The practical experience addressed how to take blood from the consenting students, how to operate the Immunochromatographic Test (ICT) and how to use smart phones to collect information. On the last day of the training, all team members visited primary school in Addis Ababa to practice the confirmatory mapping tool. Following the training, teams composed of 2 laboratory technicians and one nurse per team were formed and deployed to the selected woredas. When the central teams reached the woredas, a local guide, teacher and school director were selected to join the team.

Upon arriving at a selected school, the purpose of the study was explained to the school director. After the director’s permission was obtained, school information and global positioning system (GPS) coordinates were collected. With the help of the school director, the students were gathered at central point in the school compound. The purpose of the study was explained to the students in their local language using a poster showing the clinical manifestations of LF. Questions from students, teachers and the director were addressed by the field team.

Parental consent forms were distributed among the targeted grade students, with a request to bring them back the next day. This activity was repeated in at least two schools in a single day, based on the distance from one school to the next. The following day, students in the targeted grades with signed consent forms were lined up and randomly selected based on the sampling interval provided to that specific school by the Survey Sample Builder. Each selected student in the targeted grades, including those aged older than 14 years, participated in the study. The selected children were assigned a unique ID using barcode labels and basic demographic information from each was collected.

### Specimen collection and quality control

All data collection was done in the selected primary schools. Approximately 100 μl of whole blood was taken by finger prick from each selected child. The ICT card required 100ul of whole blood, which was collected directly from the finger using a calibrated capillary tube and added to the sample pad of the ICT card according to the WHO guidelines[10]. Cold chain was maintained to transport the ICT cards to the study sites. The ICT tests were conducted in the schools at the time of data collection, and the results were read after ten minutes. All positive ICT tests were immediately followed up by a repeat ICT test to confirm the result, which required an additional finger prick. Similarly, when the test result was invalid or indeterminate, additional blood was collected from the child to repeat the ICT test. If the child did not consent to the second blood draw the test result was recorded as invalid. The sample collection and testing continued until all selected children were tested, regardless of the number of positive results.

### Timing

The pilot study was conducted in two phases. Phase I was conducted from December 2014 to January 2015, while Phase II took place from December 2015 to March 2016. The study was designed to allow field teams to complete sampling in two schools per day. While this goal was met in most schools, in some schools, the survey took 2 days or more. On average, data collection in each woreda took 32 days to complete, including weekends during which no data collection could take place. The total survey took three months to complete due to delays in data collection in a few woredas due to logistical issues such as difficulties accessing remote communities and temporary insecurity in the woredas.

### Data management and analysis

Data was collected electronically, using Smart Phones [15]. EPHI's local server in Addis Ababa was used to retrieve data from the field and encoded later for analysis. Data verification, cleaning and descriptive analysis was done by the EPHI team and the NTD Support Center at the Task Force for Global Health using Excel and STATA statistical software. GIS software (ArcMap 10.5, ESRI) was used for mapping.

### Ethical considerations

This study obtained ethical approval from the Ethiopian Public Health Institute Scientific Ethical Review Committee (EPHI_6.13/58). Only children who came with signed consent of their parents/guardians were included in the study. All children who tested positive were referred to the nearest health center for treatment. In woredas in which the number of positives was more than the determined threshold, the recommendation was for the entire population to be treated with ivermectin and albendazole by MDA.

## Results

### Characteristics of study participants

A total of 18,254 children in 1,191 schools were tested for CFA. Females composed 45.8% (n = 8,363) of the total study participants. Of the total children in the targeted grades who are included in this survey, 92.9% (n = 16,958) were between the age 9 and 14, 7.09% (n = 1,294) were older than 14 years and 0.01% (n = 2) were aged 8 years. The mean age was 12.6 years, with 8 and 25 years the minimum and maximum ages respectively. The fewest woredas were selected in the Harari region (n = 1, 1.1% of the total sample size), and the most woredas from Oromia region (n = 19, 38.3% of the sample size). Most of the children (91%) had lived in the study area for more than ten years. Only 161 (0.88%) lived in their current location for fewer than five years.

### Circulating filarial antigen positivity

**Table 1:** List of nine woredas with CFA positive children.

Region	Woreda	Sample tested	Total CFA positive	Male	Female	Critical Cutoff	Pass/Fail
Amhara	Semada	423	4	3	1	3	Fail
Amhara	Tach Gaynt	461	5	4	1	3	Fail
Oromia	Bule-Hora	471	2	2	0	3	Pass
Oromia	Chora-Botor	477	1	0	1	3	Pass
Oromia	Miyoo	268	1	0	1	2	Pass
Oromia	G-Ayana	472	2	2	0	3	Pass
SNNP	Debub Ari	478	10	3	7	3	Fail
Tigray	Adwa	463	2	0	2	3	Pass
Tigray	Gulomekheda	463	1	1	0	3	Pass

In 36 of the 45 woredas surveyed, no children tested positive for CFA by ICT. In the 9 remaining woredas there were 28 CFA-positive results (0.15% positivity overall). The number of children testing CFA-positive ranged from one to ten per woreda, with 9 (32.1%) in two woredas in Amhara, 6 (21.4%) in four woredas in Oromia, 10(35.7%) in one woreda in SNNP and 3(10.7%) in two woredas of the Tigray region. Thirteen of the 28 positive children (46.4%) were female (Table 1). The average age of the positive children was 12.6 (range: 10–14). None of the CFA-positive children was from schools where more than five percent of the children lived in the area for five years or less. All of the CFA-positive children had lived in the current location for 10 and more years.

**Fig 2:**
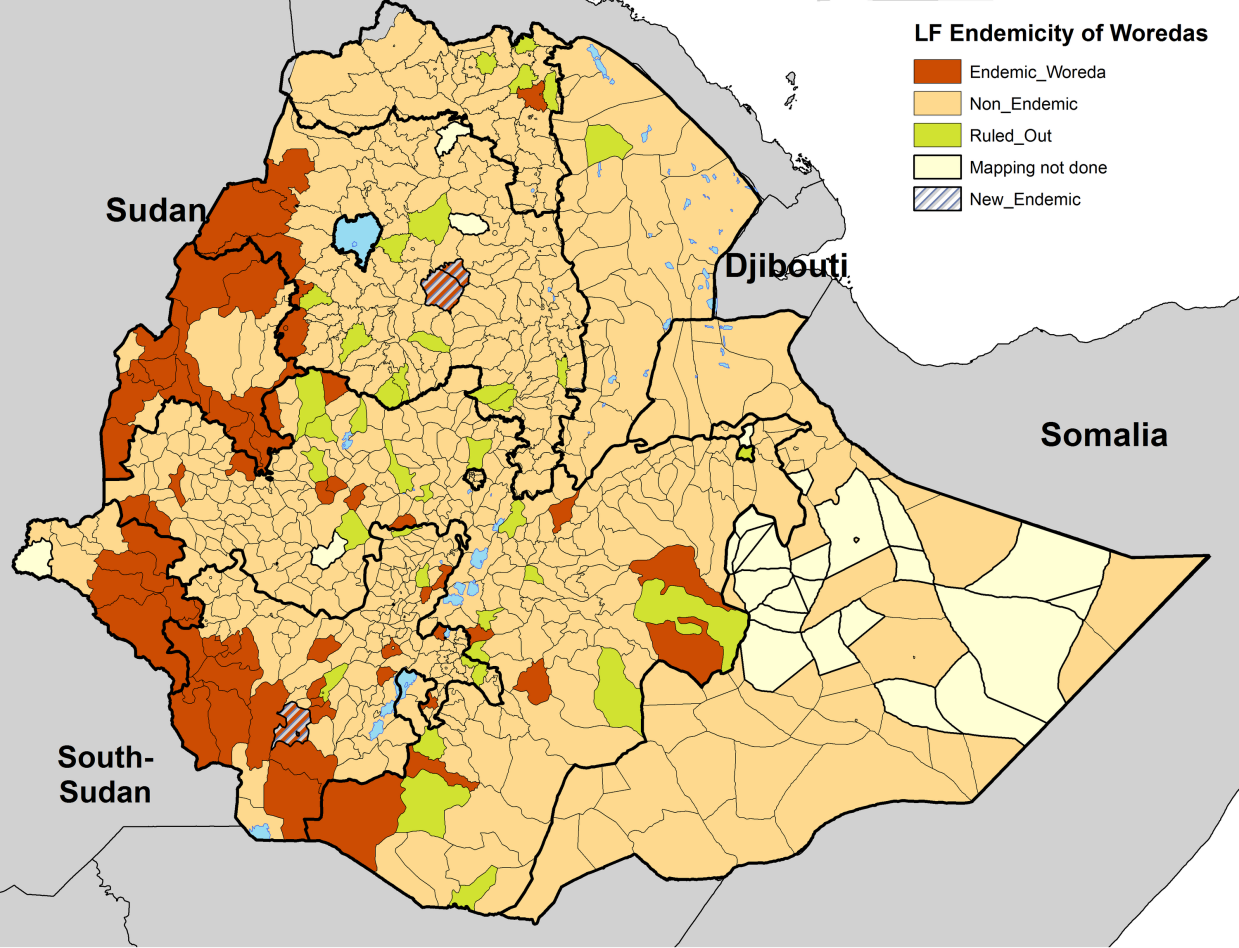
Recent map showing revised LF-endemic woredas in Ethiopia, 2015. 67 woredas already confirmed to be endemic are shown in brown. Three woredas confirmed to be endemic with this confirmatory mapping are shown in stripe line. Non-Endemic woredas as per the confirmatory mapping shown in green are 42 and Non-endemic woredas as per the previous mapping are shown in yellow. Unmapped 58 woredas are shown in greyish_yellow. www.gadm.org/ is the source of the administrative boundaries.

In 3 of the woredas: Semada, Tach Gaynt and Debub Ari, the number of CFA-positive results exceeded the maximum threshold set by the confirmatory mapping tool. As a result, these woredas were declared ‘endemic’ and in need of MDA. In the remaining six woredas where CFA-positive children were found, the CFA-positive number was less than the maximum threshold set by the confirmatory mapping tool. These woredas, together with the 36 woredas that had no positive results, were classified as ‘non-endemic’ and therefore not in need of MDA (Fig 2).

Regarding ICT test performance, 22 tests were “invalid” (0.1%), and one test was recorded as “indeterminate”. The main reason cited for the invalid tests was the lack of a control line and the child was not willing to allow the second blood draw for re-testing. There was one instance of a child having an initial “weak positive” result by ICT that, upon repeat testing, was found to be negative.

## Discussion

This manuscript describes the piloting of a new LF confirmatory mapping tool that could help NTD programs make treatment decisions in areas where the traditional WHO mapping protocol for LF has yielded uncertain results.

In 42 of the 45 woredas of uncertain endemicity, the results of the confirmatory mapping tool indicate that LF is non-endemic and no MDA is necessary. In the other 3 woredas, LF was confirmed to be endemic, and these woredas will need to initiate MDA. Only one of these 3 woredas, South-Ari, that was found to be endemic using the confirmatory mapping tool is surrounded by woredas identified as endemic in 2013 by the WHO protocol (Benatsemay, Selemago, and Teltele). The other 2 woredas found to be endemic by the confirmatory mapping tool: Simada and Tach-Gayint, are not geographically adjacent to any other woredas determined to be endemic in 2013, but they are adjacent to each other.

During the 2008 to 2013 mapping of LF the total number of woredas confirmed to be endemic or potentially endemic, including the 45 woredas with uncertain transmission, was 112[12]. The present study shrank the number of LF-endemic woredas from 112 to 70 in Ethiopia (Figs 1 and 2), dramatically decreasing the estimated number of people at risk for LF transmission and requiring MDA from 11,580,010 [12] to 5,893,309. Reducing the total number of woredas requiring MDA from 112 to 70 has major resource and logistic implications for the national LF program in Ethiopia. The cost required to implement 5 years of MDA, including the monitoring and evaluation requirements, is much greater than the cost of conducting the confirmatory mapping tool to confirm whether or not the woreda is truly endemic. The approximate cost of implementing the confirmatory mapping tool in one woreda is $7,910; a detailed description of the cost effectiveness of this survey is presented by Gass et.al. [14].

The new confirmatory mapping tool addresses several of the weakness of the standard WHO LF mapping protocol that are exacerbated in low-prevalence settings. These include restricting the age group to a population that is more likely to be indicative of recent transmission (e.g. 9–14 year olds) and expanding the number of sites sampled per woreda [14]. This latter improvement was particularly important to Ethiopia. In the current WHO mapping protocol for LF, two sites are selected based primarily on the presence of lymphedema patients. However, in Ethiopia, podoconiosis, another major cause of lymphedema, is prevalent and could be mistaken for LF-related morbidity [16]. The rationale behind the confirmatory mapping tool is similar to that of the LF transmission assessment survey (TAS), in that both employ cluster sampling of children in schools and use a 2% threshold for decision-making [10]. The two surveys differ, however, in that the sample size of the confirmatory mapping tool is approximately a fourth the size required for the TAS. As a result, the confirmatory mapping tool has less power than the TAS and, consequently, is more likely to recommend MDA in areas where the true prevalence may be below 2%. This feature was determined to be acceptable for mapping, where elimination is the ultimate goal, because it biases programs in favor of starting MDA.

Our experience demonstrates that the confirmatory mapping tool is beneficial to the national program. The survey was conducted in collaboration with both regional health and education bureaus, which greatly facilitated the survey in the selected woredas and schools. The average time to complete data collection in a single woreda was 32 days, inclusive of weekends. The implementation took longer than originally anticipated due to the need for parental consent forms and the time required to travel between some of the remote school sites. Where written parental consent is not required, we expect the total time required to be much less. Road access between schools was another challenge that caused increased number of days per woreda. If the sample size per school increased up to 50, which is similar to the sample size in the transmission assessment survey (TAS)[10], the number of schools to be visited in the woreda will be decreased. This has the advantage of decreasing the total number of days spent to complete data collection in a single woreda. Conducting the survey in schools made the data collection simple by reducing the effort required to find the target population and facilitating probability sampling.

A challenging aspect of this confirmatory mapping tool was the need for accurate school enrollment figures in advance in order to select the study sites and develop the sampling intervals. In Ethiopia, this information was obtained from the Federal Ministry of Education and, though enrollment estimation was sufficiently accurate in many woredas, there were some schools in which the enrollment figures were vastly different from the actual number of students present on the day of the study. Such discrepancies affected the accuracy of the sampling interval and resulted in sample sizes that differed dramatically from the target of 16 children per school. This issue was further compounded by an absentee rate that varied widely across the woredas. In areas with political instability or areas where there was no water [17], the absentee rate was much higher than the 15% rate assumed during the sample size calculations. In contrast to this, there were also some schools with absentee rates less than 5%. Despite these logistical challenges, the required sample size was obtained from most of the schools.

An additional challenging aspect of this survey was getting a consent from the child for the second blood draw, as the tests needed to be repeated if the result was either invalid or positive during the first blood draw. Some children were unwilling to allow a second blood draw and the test results were considered as invalid. This might lead to recording a positive result as invalid. Fortunately, the second test was conducted for all positive children in the study, so there was no risk of reporting false positive cases.

From the point of view of donors and the NTD control program of Ethiopia, the confirmatory mapping tool could be considered to be a wise investment because it reduced the need for an expensive MDA program lasting five years in the 42 ruled out woredas.

## Supporting information

S1 Table Results from the previous mapping indicates the sample size, number of sites tested and number of positives in each woreda. (DOCX)

S2 Table Descriptive overview of the woredas and confirmatory mapping results. (DOCX)

S1 Dataset Demographic information of study participants and geographic locations of each school in the study woredas. (XLSX)
