# Supplementary material for: Results of a confirmatory mapping tool for Lymphatic filariasis endemicity classification in areas where transmission was uncertain in Ethiopia
Source: PLoS Negl Trop Dis. 2018 Mar 26;12(3):e0006325. doi: 10.1371/journal.pntd.0006325 (PMC5886699; doi:10.1371/journal.pntd.0006325)
Supplement: S1 Table — (DOCX) [file pntd.0006325.s001.docx]

| **Region** | **Zone** | **Woreda Name** | **Woreda Population** | **No of Kebele tested** | **Number Surveyed** | **Number ICT Positive** |
| --- | --- | --- | --- | --- | --- | --- |
| Tigray | Central Tigray | Adwa wereda | 102203 | 2 | 198 | 1 |
| Tigray | Eastern Tigray | Atsbi Wonberta | 133411 | 2 | 202 | 1 |
| Tigray | Eastern Tigray | Gulo Meheda | 100755 | 2 | 199 | 1 |
| Tigray | Eastern Tigray | Hawzen | 141035 | 2 | 197 | 1 |
| Afar | Zone 2 | Erebti | 42,818 | 2 | 200 | 1 |
| Amhara | South Gonder | Ebinat | 248628 | 2 | 196 | 1 |
| Amhara | South Gonder | Fogera | 254975 | 2 | 198 | 1 |
| Amhara | South Gonder | Tach Gayint | 114696 | 2 | 199 | 1 |
| Amhara | South Gonder | Simada | 256789 | 2 | 200 | 1 |
| Amhara | North Shewa | Merhabete | 142310 | 2 | 198 | 1 |
| Amhara | North Shewa | Moretena Jiru | 104535 | 2 | 199 | 1 |
| Amhara | North Shewa | Yifratana Gidim | 124297 | 2 | 200 | 1 |
| Amhara | East Gojam | Aneded | 102617 | 2 | 197 | 1 |
| Amhara | East Gojam | Baso Liben | 154687 | 2 | 199 | 1 |
| Amhara | East Gojam | Enarj Enawuga | 186549 | 2 | 200 | 1 |
| Amhara | West Gojam | Jabi Tehinan | 201024 | 2 | 194 | 1 |
| Amhara | Awi | Dangila | 177102 | 2 | 194 | 1 |
| Amhara | Awi | Guangua | 250956 | 2 | 200 | 1 |
| Oromia | East Wellega | G/Ayana | 188,940 | 2 | 196 | 1 |
| Oromia | East Wellega | Sibu Sire | 124839 | 2 | 200 | 1 |
| Oromia | Jimma | Chora Boter | 111444 | 2 | 199 | 1 |
| Oromia | West Shewa | Ambo Town | 66,646 | 2 | 197 | 1 |
| Oromia | East Shewa | Adama | 190950 | 2 | 200 | 1 |
| Oromia | Arsi | Tena | 82,056 | 2 | 202 | 1 |
| Oromia | Bale | Gura Damole | 36,341 | 2 | 200 | 1 |
| Oromia | Bale | Seweyna | 80,076 | 2 | 199 | 1 |
| Oromia | Borena | Bule Hora | 326,271 | 2 | 204 | 1 |
| Oromia | Borena | Meyo | 62,133 | 2 | 200 | 1 |
| Oromia | Borena | Yabelo Town | 23,531 | 2 | 200 | 1 |
| Oromia | South West Shewa | Dawo | 105,325 | 2 | 201 | 1 |
| Oromia | South West Shewa | Goro | 56,276 | 2 | 199 | 1 |
| Oromia | West Arsi | Kokosa | 175,184 | 2 | 198 | 1 |
| Oromia | West Arsi | Kore | 126,385 | 2 | 200 | 1 |
| Oromia | West Arsi | Wendo | 106271 | 2 | 201 | 1 |
| Oromia | Horogudru | Abay Chomen | 60,420 | 2 | 186 | 1 |
| Oromia | Horogudru | Abe Dengoro | 81,351 | 2 | 191 | 1 |
| Oromia | Finfine Zuria | Sululta | 16,509 | 2 | 199 | 1 |
| SNNP | Gurage | Kebena | 62,805 | 2 | 201 | 1 |
| SNNP | Sidama | Arbegona | 176,268 | 2 | 200 | 1 |
| SNNP | Sidama | Bensa | 306,692 | 2 | 200 | 1 |
| SNNP | South Omo | South Ari | 234,373 | 2 | 202 | 1 |
| SNNP | Gamo Goffa | Denibu Gofa | 99,138 | 2 | 200 | 1 |
| SNNP | Gamo Goffa | Sawula Town | 28,547 | 2 | 200 | 1 |
| SNNP | Silti | Hulbarag | 97,688 | 2 | 200 | 1 |
| Hareri | Hareri Special Zone | Aboker | 17,175 | 2 | 201 | 1 |

**S1_Table1**: Results from the previous mapping indicates the sample size, number of sites tested and number of positives in each woreda.
