# Supplementary material for: Results of a confirmatory mapping tool for Lymphatic filariasis endemicity classification in areas where transmission was uncertain in Ethiopia
Source: PLoS Negl Trop Dis. 2018 Mar 26;12(3):e0006325. doi: 10.1371/journal.pntd.0006325 (PMC5886699; doi:10.1371/journal.pntd.0006325)
Supplement: S2 Table — (DOCX) [file pntd.0006325.s002.docx]

**S2 Table 2. Descriptive overview of the woredas and confirmatory mapping results**

| **Zone** | **Woreda Name** | **Woreda population** | **Total schools** | **No. schools selected** | **No. schools data collected** | **No. children surveyed** | **Mean age (SD)** | **No. female (%)** | **No. living < 5 years in current location (%)** | **No. ICT-positive (%)** | **Woreda’s status for For LF** |
| --- | --- | --- | --- | --- | --- | --- | --- | --- | --- | --- | --- |
| **Total** |  | **5,941,085** | **2016** | **1227** | **1191** | **18254** | **12.62 (1.59)** | **8363 (45.81%)** | **161 (0.88%)** | **28 (0.15%)** |  |
| Arsi | Xanna | 85022 | 32 | 32 | 32 | 332 | 12.93 (2.11) | 168 (50.60%) | 0 | 0 | Not Endemic |
| Awi | Dangla Zuria | 150255 | 60 | 30 | 29 | 451 | 13.15 (1.69) | 218 (48.34%) | 0 | 0 | Not Endemic |
| Awi | Guangua | 136284 | 53 | 30 | 30 | 502 | 12.57 (1.66) | 256 (51.00%) | 1 (0.20%) | 0 | Not Endemic |
| Bale | Gura Dhaamolee | 36954 | 23 | 23 | 23 | 237 | 12.79 (2.17) | 98 (41.35%) | 0 | 0 | Not Endemic |
| Bale | Sawweena | 84164 | 39 | 39 | 39 | 408 | 12.63 (1.38) | 135 (33.09%) | 0 | 0 | Not Endemic |
| Borena | Bule-Hora | 290860 | 83 | 30 | 31 | 471 | 11.97 (1.46) | 183 (38.85%) | 1 (0.21%) | 2 (0.42%) | Not Endemic |
| Borena | Miyoo | 65661 | 20 | 20 | 19 | 268 | 11.89 (1.89) | 117 (43.66%) | 0 | 1 (0.37%) | Not Endemic |
| Borena | Yaaballo | 25064 | 7 | 7 | 7 | 115 | 13.32 (2.01) | 48 (41.74%) | 0 | 0 | Not Endemic |
| Central Tigray | Adwa (Rural) | 112077 | 52 | 30 | 30 | 463 | 12.3 3(1.5) | 216 (46.65%) | 0 | 2 (0.43%) | Not Endemic |
| East Showa | Adama | 183503 | 68 | 30 | 30 | 484 | 12.7 (1.28) | 230 (47.52%) | 0 | 0 | Not Endemic |
| East Wollega | G-Ayana | 135693 | 51 | 30 | 28 | 472 | 12.26 (1.60) | 212 (44.92%) | 2 (0.42%) | 2 (0.42%) | Not Endemic |
| East Wollega | Sibu-Sire | 131385 | 52 | 30 | 30 | 468 | 12.91 (1.46) | 240 (51.28%) | 74 (15.81%) | 0 | Not Endemic |
| Eastern Tigray | Atsbi Wenberta | 132830 | 45 | 30 | 30 | 463 | 13.38 (2.12) | 245 (52.92%) | 0 | 0 | Not Endemic |
| Eastern Tigray | Gulomekheda | 100316 | 48 | 30 | 29 | 463 | 12.87 (2.03) | 221 (47.73%) | 0 | 1 (0.22%) | Not Endemic |
| Eastern Tigray | Hawzen | 127445 | 48 | 30 | 30 | 480 | 12.41 (2.19) | 244 (50.83%) | 0 | 0 | Not Endemic |
| Finfine Zuria | Sululta | 148700 | 66 | 30 | 27 | 447 | 12.49 (1.49) | 210 (46.98%) | 0 | 0 | Not Endemic |
| Gamo-Gofa | Demba Gofa | 98891 | 53 | 30 | 30 | 481 | 12.42 (1.60) | 212 (44.07%) | 0 | 0 | Not Endemic |
| Gamo-Gofa | Sawla | 32599 | 6 | 6 | 5 | 281 | 12.82 (1.36) | 122 (43.42%) | 0 | 0 | Not Endemic |
| Gurage | Kebena | 64464 | 25 | 25 | 23 | 345 | 12.77 (1.62) | 149 (43.19%) | 0 | 0 | Not Endemic |
| Harari | Aboker | 18784 | 6 | 6 | 5 | 194 | 11.9 (1.52) | 81 (41.75%) | 0 | 0 | Not Endemic |
| Horoguduru | Abbaay Cooman | 62849 | 27 | 27 | 27 | 332 | 12.2 (0.99) | 136 (40.96%) | 0 | 0 | Not Endemic |
| Horoguduru | Abbee Dangoorooo | 85445 | 31 | 31 | 31 | 320 | 12.6 (0.68) | 118 (36.88%) | 0 | 0 | Not Endemic |
| Jimma | Cooraa Botor | 116569 | 59 | 30 | 30 | 477 | 12.55 (1.39) | 225 (47.17%) | 0 | 1 (0.21%) | Not Endemic |
| Misrak Gojjam | Aneded | 104705 | 40 | 30 | 24 | 375 | 12.4 (1.76) | 208 (55.47%) | 0 | 0 | Not Endemic |
| Misrak Gojjam | Baso-Liben | 163454 | 53 | 30 | 30 | 467 | 12.01 (1.60) | 223 (47.75%) | 0 | 0 | Not Endemic |
| Misrak Gojjam | Inarj-Inawuga | 201229 | 58 | 30 | 29 | 459 | 12.02 (1.29) | 202 (44.01%) | 0 | 0 | Not Endemic |
| North Shewa | Efrata- Gidim | 105486 | 55 | 30 | 30 | 459 | 12.7 (1.93) | 222 (48.37%) | 0 | 0 | Not Endemic |
| North Shewa | Merahibete | 140047 | 35 | 35 | 34 | 522 | 12.18 (1.51) | 276 (52.87%) | 0 | 0 | Not Endemic |
| North Shewa | Moret Jiru | 108042 | 38 | 38 | 38 | 446 | 12.94 (1.79) | 215 (48.21%) | 3 (0.67%) | 0 | Not Endemic |
| S/W/Shewa | Daawoo | 92880 | 27 | 27 | 27 | 379 | 12.61 (2.08) | 155 (40.90%) | 0 | 0 | Not Endemic |
| S/W/Shewa | Goro | 58321 | 26 | 26 | 26 | 328 | 13.17 (1.11) | 166 (50.61%) | 0 | 0 | Not Endemic |
| Sidama | Arbegona | 168592 | 51 | 30 | 30 | 478 | 12.53 (1.17) | 207 (43.31%) | 3 (0.86%) | 0 | Not Endemic |
| Sidama | Bensa | 310951 | 63 | 30 | 30 | 631 | 12.28 (1.29) | 293 (46.43%) | 2 (0.32%) | 0 | Not Endemic |
| Silti | Hulbareg | 98885 | 25 | 25 | 25 | 288 | 13.35 (1.69) | 108 (37.50%) | 0 | 0 | Not Endemic |
| South Gonder | Ebinat | 256548 | 90 | 30 | 28 | 447 | 11.86 (1.49) | 209 (46.76%) | 1 (0.22%) | 0 | Not Endemic |
| South Gonder | Fogera | 252718 | 61 | 30 | 30 | 477 | 15.88 (1.54) | 222 (46.54%) | 2 (0.42%) | 0 | Not Endemic |
| South Gonder | Semada | 258964 | 116 | 30 | 27 | 423 | 12.3 (1.84) | 191 (45.15%) | 0 | 4 (0.95%) | Endemic |
| South Gonder | Tach Gaynt | 114958 | 44 | 30 | 30 | 461 | 12.5 (1.39) | 218 (47.29%) | 0 | 5 (1.08%) | Endemic |
| South Omo | Debub Ari | 236390 | 66 | 30 | 30 | 478 | 12.78 (2.15) | 202 (42.26%) | 61 (12.76%) | 10 (2.09%) | Endemic |
| West Arsi | Kokkossaa | 183933 | 43 | 30 | 31 | 475 | 11.58 (1.83) | 196 (41.26%) | 0 | 0 | Not Endemic |
| West Arsi | Qoree | 132502 | 40 | 30 | 30 | 541 | 13 (1.11) | 298 (55.08%) | 0 | 0 | Not Endemic |
| West Arsi | Wondoo | 109259 | 21 | 21 | 20 | 311 | 12.16 (1.70) | 140 (45.02%) | 0 | 0 | Not Endemic |
| West Gojam | Jabi_Tahnan | 215117 | 81 | 30 | 29 | 459 | 13.12 (1.28) | 230 (50.11%) | 3 (0.65%) | 0 | Not Endemic |
| West Showa | Amboo | 137689 | 19 | 19 | 8 | 127 | 12.6 (1.36) | 52 (40.94%) | 0 | 0 | Not Endemic |
| Zone 2 | Erebti | 64601 | 10 | 10 | 10 | 269 | 12.73 (1.68) | 46 (17.10%) | 8 (2.97%) | 0 | Not Endemic |
